# Targeting the immune milieu in gastrointestinal cancers

**DOI:** 10.1007/s00535-020-01710-x

**Published:** 2020-08-03

**Authors:** Fiona Turkes, Justin Mencel, Naureen Starling

**Affiliations:** Department of Medicine, Royal Marsden Hospital NHS Foundation Trust, London, UK

**Keywords:** Gastrointestinal cancer, Immunotherapy, Tumor microenvironment, Immune milieu

## Abstract

Gastrointestinal (GI) cancers are among the most common and lethal solid tumors worldwide. Unlike in malignancies such as lung, renal and skin cancers, the activity of immunotherapeutic agents in GI cancers has, on the whole, been much less remarkable and do not apply to the majority. Furthermore, while incremental progress has been made and approvals for use of immune checkpoint inhibitors (ICIs) in specific subsets of patients with GI cancers are coming through, in a population of ‘all-comers’, it is frequently unclear as to who may benefit most due to the relative lack of reliable predictive biomarkers. For most patients with newly diagnosed advanced or metastatic GI cancer, the mainstay of treatment still involves chemotherapy and/or a targeted agent however, beyond the second-line this paradigm confers minimal patient benefit. Thus, current research efforts are concentrating on broadening the applicability of ICIs in GI cancers by combining them with agents designed to beneficially remodel the tumor microenvironment (TME) for more effective anti-cancer immunity with intention of improving patient outcomes. This review will discuss the currently approved ICIs available for the treatment of GI cancers, the strategies underway focusing on combining ICIs with agents that target the TME and touch on recent progress toward identification of predictors of sensitivity to immune checkpoint blockade in GI cancers.

## Introduction

Gastrointestinal cancers are a huge global health problem. In 2018 almost 5 million new cases were diagnosed [1], the vast majority of which would have been at an advanced stage. While efforts to improve early diagnosis of these diseases are gaining momentum, gastrointestinal (GI) cancers are still the most deadly of all malignancies with colorectal, stomach and liver cancers representing the second, third and fourth leading causes of cancer-related deaths, respectively [1].

Over the past decade immunotherapy has revolutionized cancer treatment. Immune checkpoint inhibitors (ICIs) are now the cornerstone of managing malignancies such as lung, renal and skin cancers, among others. In some cases patients are experiencing marked radiological responses which may be sustained for years and side effects can be minimal compared with traditional chemotherapies. For an increasing number, this has meant a better quality and longer life. However, the activity of immunotherapeutic agents in GI cancers has, on the whole, been much less striking. Furthermore, in a population of ‘all-comers’, it is frequently unknown who may benefit due to the relative lack of reliable predictive biomarkers. For the majority of patients with newly diagnosed advanced/metastatic GI cancer, the mainstay of treatment still involves chemotherapy and/or a targeted agent. However, chemotherapy and/or targeted agents beyond the second-line have minimal efficacy in these diseases. More efficacious treatments are urgently needed.

ICIs work by inhibiting the interaction between an immunosuppressive ligand or signal and their corresponding receptor or protein on immune or tumor cells, thus restoring the pre-existing host immune response against cancer [2]. Examples of ICIs include those that target the PD-1:PD-L1 interaction and reinvigorate cytotoxic T cell (CTL) function mainly in peripheral tissues e.g. pembrolizumab (anti-PD-1), nivolumab (anti-PD-1), atezolizumab (anti-PD-L1) or anti-CTLA-4 agents e.g. ipilimumab which act at an earlier stage of anti-cancer immunity by encouraging T cell activation in draining lymph nodes. The tumor microenvironment (TME) is a highly complex interplay of various different innate and adaptive immune cells such as NK cells, tumor-associated macrophages (TAMs) and T and B lymphocytes, stromal cells, such as cancer-associated fibroblasts (CAFs), endothelial cells, extracellular matrix (ECM) and secreted factors and it is of critical importance to supporting the success or failure of effective ICI therapy [3]. As such, where ICIs have not been effective as single agents, research has concentrated on combining ICIs with agents that target rational components of the TME to convert a ‘cold’ TME to an ‘immunogenic’ or ‘hot’ one, thus directing the immune response toward killing cancer cells and improving patient outcomes [4].

This article will discuss the currently approved ICIs available for the treatment of GI cancers, strategies underway focusing on combining ICIs with agents that target the TME and touch on recent progress toward predictive biomarker identification. Combinations of ICIs with cancer vaccines, cellular therapies or cytokine based therapies will not be covered.

## Pre-requisites for anti-cancer immunity and the immune landscape of gastrointestinal cancers

The cancer-immunity cycle, a term first coined by Chen and Mellman in 2013, describes the seven main steps required for the immune system to carry out its final effector function of killing of cancer cells [5]. The majority of currently available ICIs work at this seventh step by blocking the PD-1/PD-L1 pathway, which usually acts as an ‘immunostat’ to prevent auto-immunity [5] but can also be up-regulated in patients with cancer [6], thereby removing the negative immune regulation on CTLs and reinstating the anti-cancer response. However if the other key events/steps in the cycle have not occurred beforehand, the anti-cancer response will not be fully potentiated by PD-1/PD-L1 blockade alone. Firstly, neoantigens are released by dying cancer cells. These are subsequently picked up by antigen presenting cells (APCs) which travel to lymph nodes to prime and activate T cells. This is a key step which requires the correct balance between co-stimulatory and inhibitory factors in the TME to promote stimulation of CTLs rather than pro-tumoral regulatory T cells (Tregs). Effector T cells then must travel to the site of the tumor, creep into the core of tumor, recognize the cancer cells on site and finally effectively kill them. In tumors which respond to single agent PD-1/PD-L1 blockade, only this final step in the sequence is flawed however, several tumor types, including most GI cancers, have other malfunctioning steps in the pathway which would also require correction.

In 2017, Chen and Mellman went on to describe three distinct immune phenotypes which could explain the mechanism of a tumour’s resistance to anti-cancer immunity [7]. So called “inflamed tumors” are most likely to respond to PD1/PD-L1 blockade as they are already permeated by plenty of different immune cells, including CTLs. The inflamed tumors might also harbor Tregs, myeloid-derived suppressor cells (MDSCs), B cells and cancer-associated fibroblasts (CAFs) which are generally inhibitory but PD-1/PD-L1 inhibitor administration is more likely to boost the CTL activity of this type of tumor than the other two “non-inflamed” phenotypes. In “immune-desert tumors”, CTLs are either a rarity or completely absent, possibly from a lack of appropriate T cell priming or activation in the lymph nodes, and in “immune-excluded tumors”, the T cells are present but they cannot get into the tumor due to inhibitory stromal or vascular factors. The Cancer Immunogram nicely plots the various factors in the immune milieu which may either be present or lacking in a patient’s tumor and thus outlines an agenda for successful individualized immunotherapy treatment [8].

Resistance to ICIs may be present from the outset or develop over a period of time on treatment due to mechanisms either intrinsic or extrinsic to the tumor cell [9, 10]. Overcoming de novo intrinsic resistance to ICIs is the main challenge in most GI cancers and may be due to the inability of the immune system to recognize cancer cells as foreign, a generally immunosuppressive TME or defective signaling pathways which would usually trigger an immune response. The first step in the Cancer Immunity Cycle requires the release of cancer neoantigens that can be recognized as non-self by the immune system [5]. It is now widely recognized that microsatellite unstable (MSI-H) or mismatch repair protein deficient (dMMR) tumors and those high tumor mutational burden (TMB) are characterized by high numbers of mutations or ‘neoantigens’ and predict sensitivity to ICIs [11–13]. However, dMMR/MSI-H GI tumors represent a very small proportion and the median somatic mutational load in these diseases is modest. Among GI cancers, oesophagogastric cancer is reported to have the highest TMB at 5 mutations/megabase, whereas pancreatic cancer has the lowest at 1 mutation/megabase [14]. Conversely, in melanoma, where ICIs exert the most efficacy, the median somatic mutational prevalence is 14 mutations/megabase [14]. Following neoantigen recoginition, antigen presenting cells e.g. dendritic cells (DCs) process the antigens and present them to T cells in lymph nodes which then become activated via major histocompatibility complex (MHC) class I or II co-stimulatory molecules. However, DCs in pancreatic cancer and cholangiocarcinoma (CCA) have been found to be limited in number or immature in advanced stages of disease [15–17], and mutations in the MHC I binding domain have been reported in colorectal cancer [18] thus potentially representing less effective antigen presentation and recognition in these tumors.

**Fig. 1:**
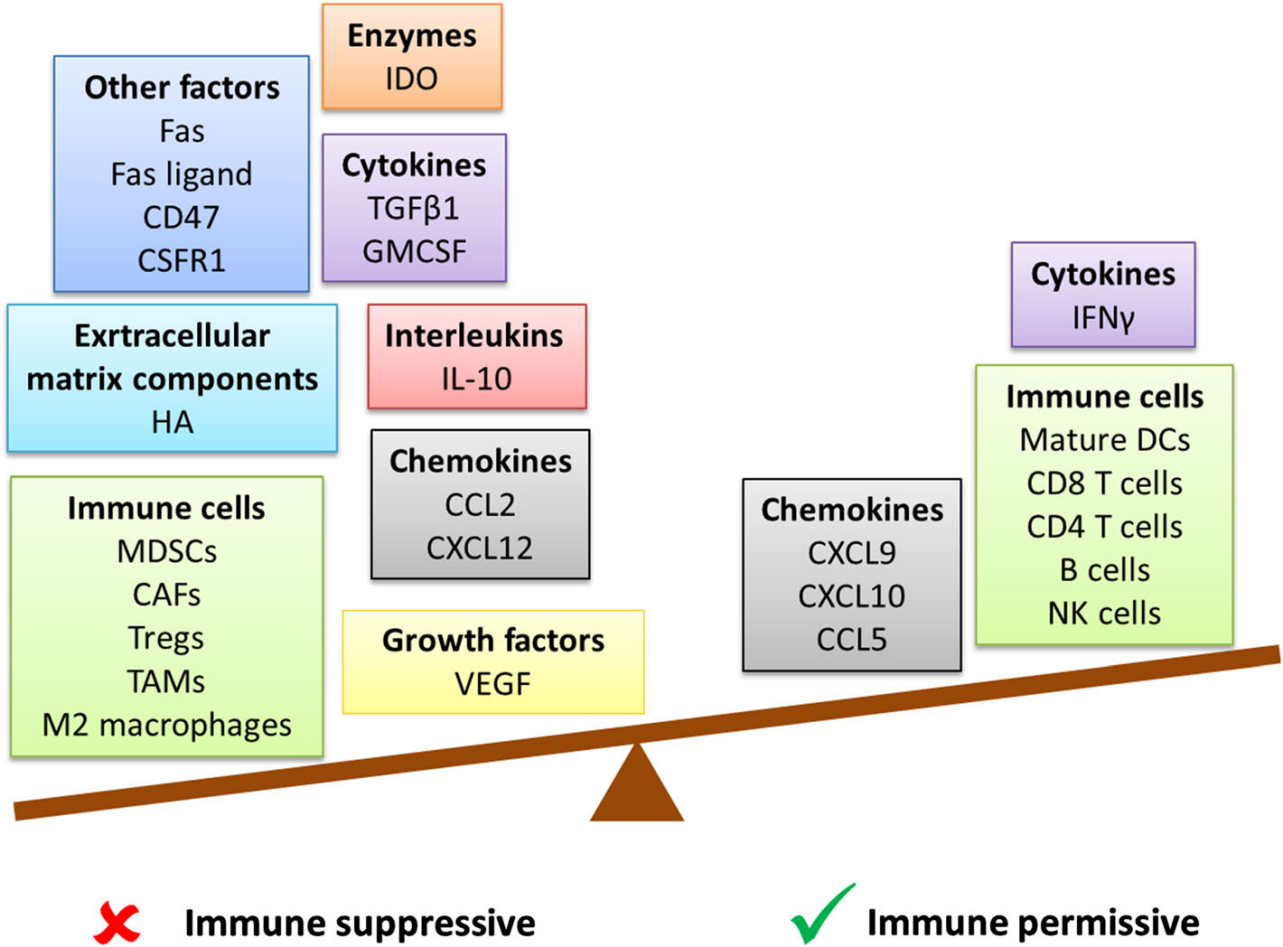
Components of the TME which favor an immune suppressive milieu generally outweigh those which are associated with the T cell inflamed phenotype and response to immune checkpoint inhibitors in gastrointestinal cancers. *CAF* cancer-associated fibroblast, *CCL* C–C motif chemokine, *CD* cluster of differentiation, *CSFR* macrophage colony-stimulating factor, *CXCL* C–X–C motif ligand, *DC* dendritic cell, *Fas* apoptosis-mediating surface antigen FAS, *GMCSF* Granulocyte macrophage colony stimulating factor, *HA* hyaluronic acid, *IDO* indoleamine 2,3-dioxygenase, *IFNγ* interferon gamma, *IL* interleukin, *MDSC* myeloid-derived suppressor cells, *NK* natural killer, *TAM* tumor-associated macrophage, *TGF-β* transforming growth factor beta, *Tregs* T regulatory cells, *VEGF* vascular endothelial growth factor

Detailed insight into TME components and their role in de novo resistance to checkpoint blockade in GI cancers has recently been reviewed by Batista et al. [19], and broadly outlined in Fig. 1. Within this article rational combinational strategies will be discussed in the context of reprogramming the TME for potentially effective ICI therapy.

## The current role of checkpoint inhibitors in gastrointestinal cancers

### Microsatellite unstable and mismatch repair deficient colorectal and other cancers

In 2017, the FDA approved both pembrolizumab and nivolumab, for use in the second-line treatment of MSI-H or dMMR metastatic colorectal cancer (mCRC) following the results of two pivotal trials. These studies demonstrated objective response rates (ORR) of 40% [20] and 32% [21] in patients with pre-treated MSI-H/dMMR mCRC who received 2 weekly pembrolizumab or nivolumab, respectively, with corresponding progression free survival (PFS) rates of 78% at almost 5 months and 50% at 12 months. In 2018, Overman et al. went onto show that giving nivolumab combined with ipilimumab every 3 weeks for 4 cycles followed by nivolumab alone to these patients, resulted in an ORR of 55% and 12 month PFS rate of 71% [22]. Accordingly the FDA granted accelerated approval to this regimen for patients with pre-treated MSI-H/dMMR mCRC in 2018. However only 3.5-5% of patients with mCRC display this MSI-H/dMMR phenotype [23, 24] and so checkpoint inhibitors currently play no role in the management of the majority of patients who have microsatellite stable (MSS) disease. Indeed the response rate to 2 weekly pembrolizumab in the MSS mCRC cohort from the aforementioned study was 0% [20].

In 2017 the FDA also granted the first tumor-agnostic approval to pembrolizumab for the treatment of any dMMR or MSI-H tumor following progression after standard treatment. The KEYNOTE-158 trial, which assessed the efficacy of 3 weekly pembrolizumab in 233 patients with 27 different types of advanced or metastatic non-colorectal MSI-H/dMMR tumors, subsequently reported an ORR of 33.4% in all patients which was durable [25]. This data reflect the findings from the earlier trials in patients with mCRC [20, 21]. However, once broken down into primary tumor type considerable variation in response rates are evident. For example, ORR was only 18.2% in patients with pancreatic cancer and 0% in patients with primary brain cancer [25] indicating that primary tumor location may influence sensitivity to ICIs even in the context of dMMR/MSI-H or that other immune suppressive factors in the TME may be in play or overall tumor burden too great [26].

### Esophagogastric cancer

The role of ICIs in esophagogastric (OG) cancers is complex and rapidly evolving, with ongoing questions as to the most appropriate timing of immunotherapy. However, there now is established evidence for ICI use, particularly PD-1 inhibitors, in these tumors (Table 2).

KEYNOTE 181 was a phase III study in patients with advanced GOJ or esophageal cancer, showing superiority of pembrolizumab over investigator choice chemotherapy, producing prolonged median overall survival (mOS) of 9.3 vs. 6.7 months in 222 patients with PD-L1 positive tumors (CPS ≥ 10) [27]. Patients with metastatic esophageal squamous cell carcinoma (SCC) also demonstrated an improved mOS (8 vs. 7 months) however in the intention to treat (ITT) cohort there was no difference in mOS between the two arms. This study led to the FDA approval of pembrolizumab in metastatic or locally advanced esophageal SCC in second or later line treatment. The KEYNOTE 061 trial involved patients with metastatic gastric or GOJ adenocarcinoma in the second-line setting who were randomized to either pembrolizumab or taxane chemotherapy [28]. In patients with CPS ≥ 1 tumors, pembrolizumab was not superior to chemotherapy in terms of mOS. However pembrolizumab demonstrated benefit in those with CPS ≥ 10 in a post hoc analysis.

Two phase Ib studies (KEYNOTE 12 and KEYNOTE 28) have evaluated the role of pembrolizumab in PD-L1 expressing gastric cancer and esophageal cancer (SCC and adenocarcinoma), respectively [29, 30]. Both studies were in the second or later line and showed impressive ORR (22% in KEYNOTE 12 and 30% in KEYNOTE 28) (Table 1). Pembrolizumab is FDA approved for use in metastatic esophagogastric adenocarcinoma (OGA) with CPS > 1 in the third or later line setting. There is little evidence to support the use of pembrolizumab in the case of PD-L1 negative OGA in the later line setting.

**Table 1:** Selected positive trials of single agent immune checkpoint inhibitor therapy in GI cancers

Trial [references]	Phase	Setting/design	Drug	*N*	Primary endpoint
Colorectal cancer					
Le et al. [20]	II	Pre-treated dMMR mCRC	Pembrolizumab	10	ORR 40%
KEYNOTE 164 [146]	II	Cohort (A):dMMR/MSI-H mCRC (≥ 2 prior lines) Cohort (B): dMMR/MSI-H mCRC (≥ 1 prior line)	Pembrolizumab	61 (A) 63 (B)	ORR 33% (A) ORR 33% (B)
CheckMate 142 [21]	II	Pre-treated dMMR mCRC	Nivolumab	74	ORR 32%
Oesophagogastric cancer					
CheckMate 032 [58]	I/II	Pre-treated advanced gastric, oesophageal, GOJ adenocarcinoma	Nivolumab	59	ORR 12%
KEYNOTE 059 [147]	II	Pre-treated advanced gastric, oesophageal, GOJ adenocarcinoma	Pembrolizumab	259	ORR^a^ 11.6%
ATTRACTION-02 [148]	III	Pre-treated advanced gastric adenocarcinoma, GOJ (≥ 2 chemotherapy lines)	Nivolumab (vs placebo)	330 (163)	Median OS 5.26 vs 4.14 (HR 0.62; *p* < 0.0001)
KEYNOTE 028 [149]	Ib	PD-L1 positive pre-treated advanced oesophageal, GOJ adenocarcinoma or squamous cell carcinoma	Pembrolizumab	23	ORR^a^ 30.4%
KEYNOTE 012 [29]	Ib	PD-L1 positive pre-treated advanced gastric or GOJ adeoncarcinoma	Pembrolizumab	39	ORR^a^ 22%
KEYNOTE 158 [25]	II	dMMR/MSI-H advanced gastric cancer	Pembrolizumab	24	ORR 45.8%
Hepatocellular carcinoma					
Sangro et al. [39]	II	Advanced HCC with chronic HCV infection	Tremelimumab	20	ORR 17.6%
CheckMate 040 [37]	II	Advanced HCC	Nivolumab	48	ORR 15% (dose escalation)
				214	ORR 20% (dose expansion)
KEYNOTE 224 [38]	II	Advanced HCC	Pembrolizumab	104	ORR 17%
Biliary tract cancer					
KEYNOTE 158 [41]	II	Advanced BTC (unselected although 61 patients were found to have PD-L1 positive tumours)	Pembrolizumab	104	ORR 5.8%
KEYNOTE 158 [25]	II	dMMR/MSI-H advanced cholangiocarcinoma	Pembrolizumab	22	ORR 40.9%
KEYNOTE 028 [40]	Ib	PD-L1 positive advanced BTC	Pembrolizumab	24	ORR^a^ 17%
Kim et al. [42]	II	Advanced BTC (unselected)	Nivolumab	45	ORR 22%
Pancreatic cancer					
KEYNOTE 158 [25]	II	dMMR/MSI-H advanced PDAC	Pembrolizumab	22	ORR 18.2%
Anal cancer					
NCI9673 [44]	II	Pre-treated advanced SCCA	Nivolumab	37	ORR 24%
KEYNOTE 028 [45]	Ib	PD-L1 positive advanced SCCA	Pembrolizumab	24	ORR^a^ 17%

*BTC* biliary tract cancer, *dMMR* deficient MisMatch Repair, *GOJ* gastro-oesophageal junction, *HCC* hepatocellular carcinoma, *HCV* hepatitis C virus, *mCRC* metastatic colorectal cancer, *MSI-H* microsatellite instability high, *ORR* overall response rate, *PDAC* Pancreatic ductal adenocarcinoma, *PD-L1* programmed death-ligand 1, *SCCA* squamous cell cancer of the anal canal

^a^Co-primary endpoint with safety

Nivolumab has efficacy in advanced gastric and GOJ adenocarcinoma in the third line setting regardless of PD-L1 expression, as seen in the ATTRACTION-2 study [31]. In this study, 493 patients from Asian countries were randomly assigned to either nivolumab or placebo, following progression on 2 or more regimens. The ORR was 11% with a survival benefit (12 month survival 27% vs. 11%). Nivolumab is approved in Japan for use in the second-line treatment of advanced gastric cancer, regardless of PD-L1 expression (Table 2). The ATTRACTION-3 study has since also confirmed efficacy for nivolumab in advanced esophageal SCC however this indication is not yet approved [32]. This study assigned 419 patients to either nivolumab or taxane chemotherapy in the second line, regardless of PD-L1 expression. Nivolumab improved mOS by an addition of almost 3 months (10.9 vs. 8.4 months) with an improved toxicity profile. Again, there was no signal to suggest PD-L1 expression impacted efficacy.

**Table 2:** Checkpoint inhibitors approved for use in GI cancers

Agent	Disease type	Indication	Approving body (year)
Pembrolizumab	dMMR/MSI-H mCRC	Relapsed/refractory	FDA (2017)
Nivolumab	dMMR/MSI-H mCRC	Relapsed/refractory	FDA (2017)
Nivolumab + ipilimumab	dMMR/MSI-H mCRC	Relapsed/refractory	FDA (2018)
Pembrolizumab	Any dMMR/MSI-H tumour type	Following progression on standard treatment	FDA (2018)
Pembrolizumab	Metastatic/advanced PD-L1 positive (CPS ≥ 1) gastric/GOJ cancer adenocarcinoma	Following progression after ≥ 3 lines of systemic therapy	FDA (2017)
Nivolumab	Metastatic/advanced gastric cancer	Following progression after chemotherapy in 3rd line setting	MHLW (2017)
Pembrolizumab	Advanced oesophageal squamous cell cancer with CPS ≥ 10	Following progression after ≥ 2 lines of systemic therapy	FDA (2019)
Pembrolizumab	Advanced HCC	Second-line (following previous treatment with sorafenib)	FDA (2018)
Nivolumab	Advanced HCC	Second-line (following previous treatment with sorafenib)	FDA (2017)
Atezolizumab + bevacizumab	Advanced HCC	First-line	FDA (2018)

*CPS* combined positive score, *dMMR* deficient MisMatch Repair, *FDA* U.S. Food and Drug Administration, *GOJ* gastro-oesophageal junction, *HCC* hepatocellular carcinoma, *mCRC* metastatic colorectal cancer, *MHLW* Japanese Ministry of Health Labour and Welfare, *MSI-H* microsatellite instability high, *PD-L1* programmed death-ligand 1

### Pancreatic cancer

Trials of single-agent immune checkpoint inhibitors have been particularly disappointing in pancreatic cancer. In a phase II study of ipilimumab monotherapy in 27 patients with advanced pancreatic ductal adenocarcinoma (PDAC), one patient had an objective response after initial pseudoprogression however there were no responses in the other 26 patients [33] Similarly, the anti-PD-L1 antibody BMS-936559 yielded 0% ORR in 14 patients with advanced PDAC in a multi-tumor type phase I study [34] Postulated reasons for such dramatic failures include low tumor mutational burden [35] and particularly dense desmoplastic stroma within the TME, impervious to adequate mobilization of immune cells [36].

### Hepatocellular carcinoma

Following favorable results from the CheckMate 040 study, the FDA granted accelerated approval to nivolumab in September 2017 (Table 2). In this phase II study 246 patients with advanced HCC were treated with nivolumab in any line and at the 3 mg/kg dose which was taken forward to the dose expansion phase, the ORR was reported as 20% and the 9 month survival rate was 74%. PD-L1 positive status did not correlate with response in this study [37] The FDA subsequently granted approval for the use of pembrolizumab in second-line treatment of advanced HCC following the results of the phase II KEYNOTE 224 study where 104 patients who were intolerant of or had progressed on sorafenib were given 3 weekly pembrolizumab [38] In this study the response rate was 17% and the 12 month overall survival rate was 54% [38] Tremelimumab (anti-CTLA-4 antibody) has also proven efficacious in patients with advanced HCC due to chronic hepatitis C infection (Table 1). Interestingly, a reduction in viral load was also noted with response to tremelimumab therapy suggestive of improved T cell immunosurveillance with checkpoint inhibition [39].

### Biliary tract cancer

Biliary tract cancers (BTCs) are rare malignancies so data from trials of checkpoint blockade in patients with BTC have come from basket trials with BTC cohorts. In KEYNOTE-028, pembrolizumab was given to patients with PD-L1 positive tumors including 24 patients with heavily pretreated PD-L1 positive BTC [40]. The ORR was 17% and responses were largely durable (over 40 weeks) [40]. In a larger study of 104 biomarker-unselected patients with BTCs (although 61 patients’ tumors did test positive for PD-L1), the response rate was only 5.8%. However responses were also durable and the 12 month OS rate was 37.8% [41] In KEYNOTE-028, dMMR/MSI-H status was not reported and in KEYNOTE-058, no tumor was MSI-H. In contrast, response rates of 41% have been reported in dMMR/MSI-high cholangiocarcinoma [25] Nivolumab has been proven to show equivalent efficacy in patients with previously treated BTCs (Table 1), retrospective PD-L1 analysis is ongoing for this study [42].

### Anal cancer

Human papilloma virus (HPV) infection has been associated with higher numbers of tumor infiltrating lymphocytes and up-regulation of PD-1 checkpoints and is attributable to over 80% of case of squamous cell carcinoma of the anal canal (SCCA) [43]. In the first phase II study of a checkpoint inhibitor trialed in patients with SCCA, 37 patients with refractory disease were treated with nivolumab, 24% of whom had a radiological response including 2 complete responses and 7 partial responses [44] Median duration of response was 5.8 months and the longest response was recorded as 10.4 months but was still continuing at the data cut-off date [44]. Archival tissue was available from 15 patients and every one was positive for HPV infection, PD-L1 expression on tumor cells was also higher in responders compared to non-responders [44]. In a subsequent phase Ib study of pembrolizumab in 24 patients with PD-L1 positive advanced SCCA, 17% achieved a radiological response and all of these patients had received a prior treatment [45]. HPV status was not collected in this study. Based on the responses demonstrated in these two trials, the latest NCCN guidelines recommend either nivolumab or pembrolizumab in the second or later line treatment of SCCA [46].

### Candidate predictors of sensitivity to immune checkpoint blockade in gastrointestinal cancers

As more and more approvals are coming through for use of ICIs in patients with GI cancers, the key research focus has been to try and improve and refine patient selection to expand the potential benefit of these therapies. In parallel to MSI-H/dMMR status discussed above, tumor TMB status, a reflection of the number of mutations within the tumor and thus the presumed neoantigen load capable of triggering an immune response [47], is an important candidate to highlight. Generally TMB is defined as number of non-synonymous somatic mutations (single nucleotide variants and small insertions/deletions) in coding regions and reported as mutations per megabase as standard [14, 48]. TMB can be measured by sequencing, if enough tissue is available, or by a panel-based approach which have demonstrated strong concordance [14, 49]. In a phase Ib/II study of toripalimab (anti-PD1) in chemo-refractory gastric cancer there was markedly superior OS in the TMB-high compared to the TMB-low group (14.6 vs. 4 months; HR 0.48) [50]. A cutoff of the top 20% of the TMB (12 mutations/Mb) was selected as defining a tumor as TMB-High (*n* = 12, 54 patients in total in the study) and patients with TMB < 12 mutations/Mb were defined as TMB-Low [50]. TMB-High cut-offs vary depending upon underlying histology and if there is a wide range of TMB values then the cut-off might potentially be set too high however the top 20% boundary has been shown to predict survival in a number of other tumor types [51]. In another study, however, the survival benefit of ICI therapy in patients with TMB-high esophagogastric cancer disappeared when those with MSI-high tumors were omitted from the group [52]. It does appear that, irrespective of histology, some patients with TMB-high tumors do not respond to ICIs and conversely there are some with TMB-low tumors who do [12, 35] and so, at present, TMB alone is not the panacea of predictive biomarkers. Of course one of the major challenges is intra-tumoral heterogeneity which means that the area of tissue which is eventually analyzed may show a very different mutational profile depending on from where in the primary or metastasis the biopsy sample has been taken [53]. Blood-based TMB (bTMB) assessment by liquid biopsy may overcome this particular hurdle and indeed a method of bTMB assessment has recently been validated in lung cancer [54].

PD-L1 expression is another important biomarker to mention, not least because a number of approved uses of ICIs in esophagogastric cancers mandate tumor PD-L1 positivity (Table 2). Historically methods of PD-L1 determination have varied widely however now most ICIs have their own companion-diagnostic for PD-L1 assessment which is generally measured by either combined positive score (CPS) [55, 56] or tumor proportion score (TPS) [57]. Nevertheless, in the ATTRACTION-02 study, the survival benefit from nivolumab in pre-treated patients with esophagastric cancer was observed in both PD-L1 positive and negative patients [58]. Furthermore, in KEYNOTE 012, where patients were subjected to repeated on-treatment biopsies, there was substantial disparity in PD-L1 expression between samples [29]. These findings suggest that PD-L1 status alone may not be the most useful predictor of successful ICI therapy in esophagogastric cancer.

Virus-associated cancers also demonstrate increased mutational loads [59, 60] and immune exhaustion [61] proposing a potential predictor of ICI efficacy. Epstein Barr Virus (EBV)-positive gastric cancers in particular have relatively high levels of CTLs and IFN-γ [62] and indeed, single agent pembrolizumab yielded an immense ORR of 100% in Korean patients with advanced EBV-positive gastric cancer [63] suggesting that EBV positivity may be an important predictive biomarker in gastric cancer. Correspondingly, the high response rates to nivolumab seen in patients with SCCA highlighted in the previous section [44] may well also have been linked to the high rates of associated HPV infection in this disease. Conversely however in the Checkmate 040 study, response rates to nivolumab did not significantly differ in patients with HCC caused by hepatitis B or C or not [37].

While the candidate predictive biomarkers so far discussed have been helpful in determining which patients with GI cancers may benefit most from ICI therapy, more precise biomarkers are clearly needed. Another potential contender on the horizon is the gut microbiome particularly in colorectal cancer where, for example, a certain species of bacterium has been shown to be inversely correlated to levels of T cell infiltrates in MSH-H CRC [64] and thus potentially an inferior outcome to ICI therapy. There may also be mileage in a composite biomarker approach given that, for example, in gastric cancer the combination of EBV and PD-L1-positivity but not MSI-H status was associated with response to checkpoint inhibition [64, 65]. Specific TME phenotypes defined by TME infiltration patterns of immune cells may also develop into a promising predictor of ICI response in gastric cancer [66].

## Combining immune checkpoint inhibitors with agents that target the TME

### Combination checkpoint blockade with chemotherapy

Traditional cytotoxic chemotherapy exerts its anti-neoplastic activity through direct cytotoxicity, impacting the cell cycle and leading to apoptosis. However, studies have shown that some chemotherapeutic agents also have immune modulating effects, through down-regulating immune inhibitory cells and stimulating the production of pro-inflammatory cytokines, leading to a ‘hot’ or T cell inflamed TME [67] (Table 3). Combining cytotoxic chemotherapy with ICIs have shown impressive outcomes in the treatment of other solid malignancies, including lung and head and neck cancers [68–70] however, thus far, studies of combined chemotherapy with ICIs in GI cancers have shown mixed outcomes.

**Table 3:** Potential immunomodulating effects of various chemotherapeutic agents on the TME

Chemotherapeutic agent	Immune response
Fluorouracil	Depletes MDSCs [150] Increases IFNy, IL1b and IL-17 production [150, 151] Activation of NLRP3 inflammasome [152]
Platinum agents	Increased IFNy, TNFα production through upregulation of CD8 T cells [153] Upregulation of HMGB-1 [154, 155] Upregulation of MHC1 expression [156]
Anthracyclines	Upregulation of HMGB-1 expression [157] Increased expression of type 1 interferons [158] Upregulation of IFNy and STING pathway [159]
Taxanes	Increased production of IFNβ [160] Formation of micronuclei DNA and activation of STING pathway [161] Increased expression of MHC class I expression [160]
Gemcitabine	Depletes circulating Tregs [162] Depletion of MDSCs [163]

*CD* cluster of differentiation, *DNA* deoxyribonucleic acid, *HMGB-1* high mobility group box 1, *IFN* interferon, *IL* interleukin, *MDSC* myeloid-derived suppressor cells, *MHC* major histocompatibility complex, *NLRP3* NACHT LRR and PYD domains-containing protein 3, *NK* natural killer, *STING* stimulator of interferon genes, *TNF-α* tumour necrosis factor alpha, *Tregs* T regulatory cells

The KEYNOTE 059 trial (*n* = 25) determined that pembrolizumab combined with platinum/fluropyrimidine in the first-line treatment of advanced gastric cancer had a manageable safety profile and, furthermore, showed impressive ORR (60%) and mOS of 13.8 months [71]. KEYNOTE 062 subsequently investigated the role of pembrolizumab with or without chemotherapy, versus chemotherapy alone in first-line, advanced OGA [72]. This study of 763 patients showed non-inferiority in mOS between pembrolizumab and chemotherapy and in an exploratory analysis of patients with PD-L1 CPS ≥ 10, there was an improved mOS with pembrolizumab (17 vs. 11 months), with a more tolerable toxicity profile. Importantly, however, combination pembrolizumab with chemotherapy was not superior to chemotherapy alone. The phase III KEYNOTE 590 study of pembrolizumab with chemotherapy as first-line therapy in advanced PD-L1 positive CPS ≥ 10 oesopheageal cancer is ongoing (NCT03189719) and will clarify whether the combination of chemotherapy plus ICI can improve OS in these patients over chemotherapy alone. Preliminary results also suggest that the combination of chemotherapy, pembrolizumab and HER2 directed therapy for HER2 amplified OGA may be efficacious. The ongoing phase II, single arm study evaluating trastuzumab, capectiabine and oxliaplatin in combination with pembrolizumab in the first-line setting of advanced HER2 amplified OGA which showed a high ORR (83%) with mPFS 11.4 months which showed a high ORR (83%) with mPFS 11.4 months [73] and is currently still recruiting (Table 4).

**Table 4:** Selected ongoing studies combining TME modulating agents with immune checkpoint inhibitors in advanced or metastatic gastrointestinal cancers

ICI partner drug/TME modulating effect	Mechanism of action	Drug	ICI	Phase	Study population	Trial identifier
Angiogensis	Multi TKI	Cabozantinib	Durvalumab	Ib	OGA, CRC, HCC	NCT03539822
	VEGFR2 antagonist	Ramucirumab	Durvalumab	I	OGA, HCC, NSCLC	NCT02572687
	Multi TKI	Lenvatinib	Pembrolizumab	II	OGA	NCT03321630
	Multi TKI	Cabozantinib	Atezolizumab	III vs sorafenib	HCC	NCT03755791
	VEGF-A inhibitor	Bevacizumab	Atezolizumab	III vs sorafenib	HCC	NCT03434379
	Multi TKI	Lenvatinib	Pembrolizmab	III vs lenvatinib	HCC	NCT03713593
	Multi TKI	Lenvatinib	Nivolumab	II	HCC	NCT03841201
	Multi TKI	Sorafenib	Nivolumab	II	HCC	NCT03439891
	Multi TKI	Regorafenib	Pembrolizumab	Ib	HCC	NCT03347292
	Multi TKI	Sorafenib	Pembrolizumab	Ib/ll	HCC	NCT03211416
	VEGF-A inhibitor	Bevacizumab	Atezolizumab	II	‘MSI-like’ mCRC	NCT02982694
	Multi TKI	Regorafenib	Pembrolizumab	I/II	mCRC	NCT03657641
Chemotherapy	TS inhibitor, DNA damaging agent	Fluorouracil, oxaliplatin	Pembrolizumab	II	mCRC (MSS and MSI)	NCT02375672
	TS inhibitor, DNA damaging agent, anti-HER2 antibody	Capecitabine, oxaliplatin, trastuzumab	Pembrolizumab	II	*HER2* positive gastric cancer	NCT02954536
ICI	Anti-LAG3 antibody	Relatlimab	Nivolumab	II	RAS/RAF WT mCRC after progression on anti-EGFR antibody	NCT03867799
Epigenetic regulation	HDAC inhibitor	Entinostat	Pembrolizumab	II	Multi inc. pMMR CRC	NCT02437136
	HDAC inhibitor	Entinostat	Nivolumab	II	CCA or PDAC	NCT03250273
	HDAC inhibitor	Domatinostat	Avelumab	IIa/IIb	MSS OGA or CRC	NCT03812796
	HDAC inhibitor	CXD101	Nivolumab	Ib/II	MSS CRC	NCT03993626
	DNMT inhibitor	Azacytidine	Durvalumab	II	Multi inc. MSS CRC	NCT02811497
	IDO inhibitor	BMS-986205	Nivolumab	I/II	HCC	NCT03695250
	IDO inhibitor	Epacadostat	Pembrolizumab	II	OGA	NCT03196232
Wnt signalling modulators	DKK1 antibody	DKN-01	Atezolizumab	IIa/IIb	MSS OGA	NCT04166721
	Porcupine inhibitor	CGX1321	Pembrolizumab	I/Ib	All GI tumours	NCT02675946
	Porcupine inhibitor	ETC-1922159	Pembrolizumab	Ia/Ib	Advanced solid tumours inc. MSS CRC	NCT02521844
	Porcupine inhibitor	LGK974	PDR001 (anti-PD-1)	I	Malignancies dependent on Wnt ligands inc PDAC, *BRAF* mutant CRC, oesophageal SCC	NCT01351103
Stromal targeting	CSF-1 antibody	Lacnotuzumab	PDR001	II	Gastric cancer	NCT03694977
	CSF-1R tyrosine kinase inhibitor	Pexidartinib	Durvalumab	I	CRC or PDAC	NCT02777710
	CSF-1R antibody	Cabiralizumab	Nivolumab	I	Advanced solid tumours inc PDAC	NCT02526017
	FAK inhibitor	Defactinib	Pembrolizumab	I/IIa	Advanced solid tumours inc PDAC	NCT02758587
	CD40 agonist + chemotherapy	APX005M + gemcitabine and nab-paclitaxel	Nivolumab	Ib/II	PDAC	NCT03214250

*CD* cluster of differentiation, *CSF-1* colony stimulating factor-1, *CSFR-1* colony stimulating factor-1 receptor, *DKK Dickkopf* related protein, *DNMT* DNA methyltransferase, *FAK* focal adhesion kinase, *GI* gastrointestinal, *HCC* hepatocellular carcinoma, *HDAC* histone deacetylase, *ICI* Immune checkpoint inhibitor, *IDO* indoleamine 2,3 dioxygenase, *mCRC* metastatic colorectal cancer, *MSI* microsatellite instability, *MSS* Microsatellite stable, *NSCLC* Non-small cell lung cancer, *OGA* oesophagogastric cancer, *PDAC* pancreatic ductal adenocarcinoma, *PD-L1* programmed death-ligand, *pMMR* proficient MisMatch Repair, *SCC* squamous cell carcinoma, *TKI* tyrosine kinase inhibitor, *TS* thymidylate synthase, *VEGF* vascular endothelial growth factor, *VEGFR* vascular endothelial growth factor receptor, *WT* wild-type

Combination ICI plus chemotherapy has also been studied in mCRC. In a phase II trial, 30 patients with mCRC, irrespective of MMR status, were treated with pembrolizumab in combination with mFOLFOX in the first-line setting, the ORR was 53% with 100% disease control rate at 8 weeks [74]. Conversely, however, the combination of trifluridine/tipiracil with nivolumab did not demonstrate clinically significant benefit in a heavily pre-treated population [75].

### Combination checkpoint blockade with anti-VEGF therapy

**Fig. 2:**
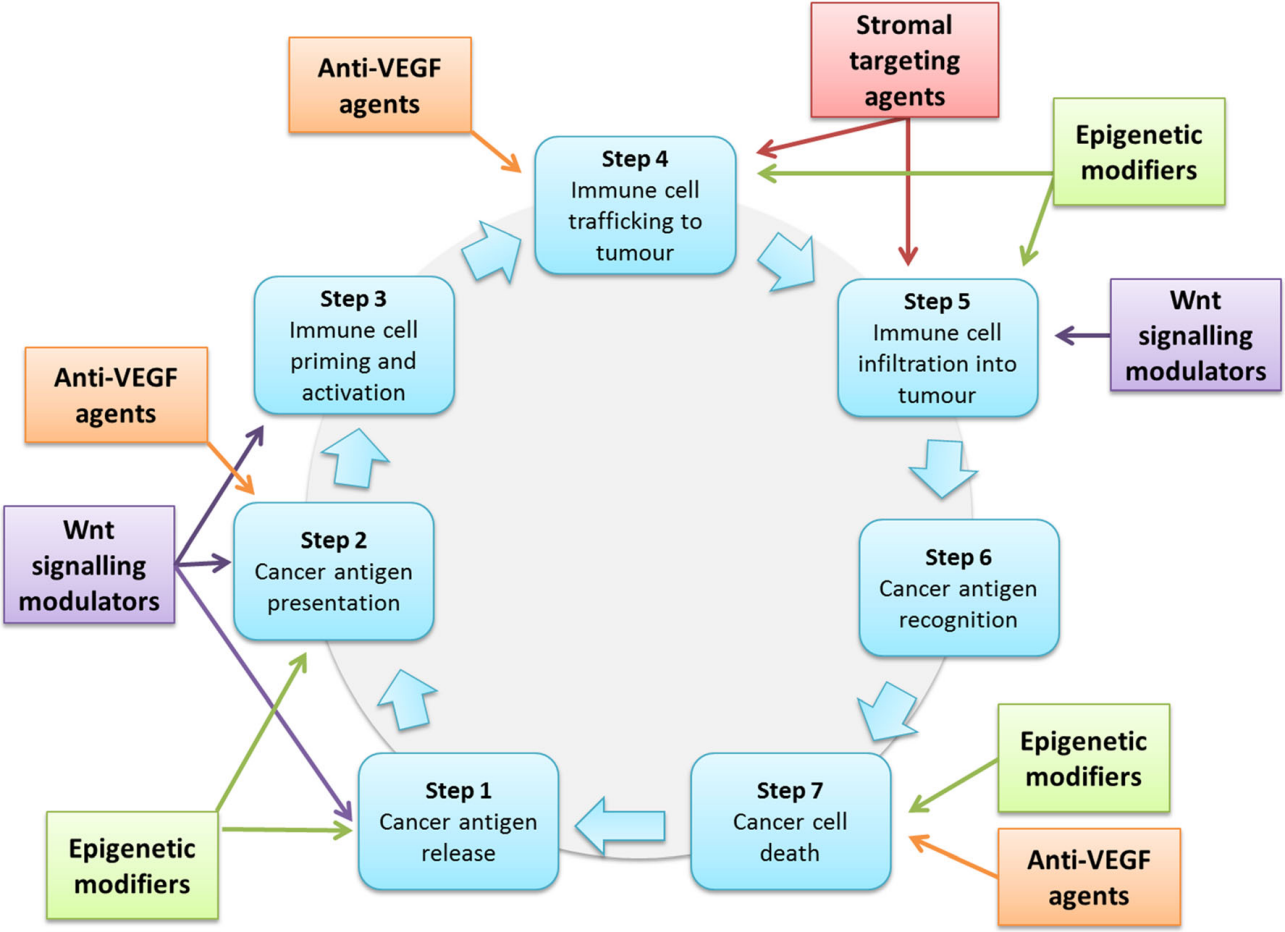
Examples of partner agents which may work in synergy with immune checkpoint inhibitors in gastrointestinal cancers by promoting progression through the cancer immunity cycle [5] at these various steps. PD-1/PD-L1 blockade alone is not sufficient to activate the anti-cancer immune response in most ‘immunologically cold’ gastrointestinal cancers but combination strategies involving agents which favorably modulate the tumor microenvironment and immune milieu may render checkpoint inhibitor therapy more efficacious in these diseases. *VEGF* vascular endothelial growth factor

Vascular endothelial growth factor (VEGF) regulates the tumoral vascular environment which enhances angiogenesis [76], and is frequently upregulated in tumors to promote growth and metastases. Blocking VEGF has anti-angiogenic effects and improves the delivery of anti-neoplastic therapies and T cell trafficking to the tumor (see Fig. 2 step 4 of cancer immunity cycle) [77]. Anti-VEGF therapies including bevacizumab in combination with chemotherapy have been shown to improve survival in mCRC [78]. There is also data to support the use of ramucirumab, a monoclonal antibody to VEGFR-2, in combination with paclitaxel in advanced OGA in the later line setting [79]. Sorafenib and lenvatinib, both with anti-VEGF activity, are also effective for treatment of advanced HCC [80].

There is evidence that VEGF also has immune-modulating effects, through impacting DCs and inhibiting T cell activity (see Fig. 2—step 2 and 7 of Cancer Immunity Cycle) [81]. Combination immunotherapy with anti-VEGF therapies have been used in the treatment of advanced renal cell cancer (RCC) [82, 83] and non-small cell lung cancer [84] with improvements in survival in these tumor types. The combination of ramucirumab and nivolumab was investigated in a phase I/II study in the second-line treatment of advanced gastric adenocarcinoma. This combination produced an ORR 27% and mOS of 9 months [85]. Furthermore, in a phase II study of 133 patients with chemo-refractory mCRC randomized to either capecitabine, bevacizumab with either atezolizumab or placebo. The addition of atezolizumab produced a non-significant improvement in mPFS (primary end point), with low ORR (8.5% vs. 4.3%) [86].

The combination of atezolizumab and bevacizumab has also demonstrated efficacy in the first-line setting of advanced HCC. The phase III IMbrave 150 study showed that this combination improves mOS compared with sorafenib and was granted FDA approved for this indication [87]. Table 4 outlines some of the currently ongoing clinical trials of anti-angiogenic agents combined with ICIs in GI cancers.

### Dual checkpoint blockade

Dual ICI therapy targeting CTLA-4 and PD-1 inhibition has established efficacy in malignancies such as melanoma [88] and RCC [89]. The proposed mechanism being that simultaneous inhibition of CTLA-4 and PD-1 provides an enhanced anti-tumor T cell response. However the role of dual checkpoint blockade in GI cancers is still being investigated. The CheckMate 142 trial has recently established a role for nivolumab and ipilimumab in dMMR mCRC. Of the 119 patients treated with this regimen, 58% had a response to treatment [90]. This combination is now FDA approved for use in dMMR/MSI-H mCRC in the second-line setting. The CheckMate 032 study investigated the role of nivolumab with ipilimumab in patients with advanced gastric, GOJ and esophageal cancers. The combination produced ORR of 8% (for nivolumab 3 mg/kg and ipilimumab 1 mg/kg) and 24% (nivolumab 1 mg/kg and ipilimumab 3 mg/kg) [58].

Relatlimab is a monoclonal antibody against the lymphocyte activation gene-3 (LAG-3) on tumor infiltrating lymphocytes (TILs). Blockade of this inhibitory signal can promote T cell mediated tumor cell death and activate TILs in CRC typically express high levels of LAG3, which make this a promising therapeutic target [91]. Furthermore, anti-EGFR antibody treatment appears to be capable of upregulating PD-1 and LAG3 checkpoints in RAS/RAF wild-type mCRC [92]. A phase II study to assess the efficacy of combination nivolumab and relatlimab in patients with RAS/RAF wild-type mCRC who develop acquired resistance to anti-EGFR treatment is currently recruiting. (Table 4).

### Combination checkpoint blockade and epigenetic modifiers

Epigenetics is the modification of gene expression without alteration of DNA’s nucleic acid sequence [93]. These modifications include DNA methylation, alterations to histone proteins and remodeling of chromatin and they are often aberrant in disease processes such as cancer [93, 94]. Notably however, alterations in the epigenome rather than the core genetic material of cells have been shown to be reversible and hence a potential anti-cancer strategy [95].

Various epigenetic therapies have been approved for use in the treatment of hematological malignancies however their use as single agents in solid tumors, including CRC, has been historically somewhat limited for reasons such as short half-lives, high toxicity or low or no responses [96]. This has led to more recent research instead using them as ‘sensitizers’ in combination with other anti-cancer therapies such as chemotherapy and targeted agents which have purported some success particularly in CRC [96, 97]. Furthermore, there is increasing evidence that epigenetic agents can promote the anti-cancer immune response via multiple mechanisms including increased antigen release and antigen presentation [steps 1 and 2 of the Cancer Immunity Cycle (Fig. 2)], T cell trafficking and infiltration into the tumor (steps 4 and 5) as well as restoring effector T cell function (step 7), suggesting potential synergy with checkpoint inhibitors particularly in immunologically ‘cold’ tumors [98].

By far the most studied epigenetic modulators are inhibitors of histone deacetylases (HDACi) and DNA methyltransferases (DNMTi). Pre-clinical testing demonstrates that the DNMTi 5-azacitadine (5-AZA) upregulated 15 gene sets involved in the immune response such as antigen presentation, interferon signaling and chemokine and cytokine signaling in colorectal cancer cell lines [99]. Mouse models of colorectal tumors were completely eradicated when 5-AZA and entinostat (a class I HDACi) were combined with PD-1 and CTLA-4 antibodies by markedly reducing immune inhibitory MSDC populations thus allowing for expansion of CD8+ T cell cohorts [100]. Entinostat also improved responses to checkpoint blockade in pancreatic cancer mouse models via the same beneficial effects on the TME [101]. Furthermore, after treatment of colorectal cancer PDX models with decitabine (DNA methyltransferase inhibitor), antigen processing and presenting genes were significantly upregulated in addition to some cytokine and chemokine-related genes such as CXCL1, cell proliferation genes were conversely downregulated and there was influx of CD4+ and CD8+ T cells into the tumor [102]. Successively the combination of decitabine and PD-1 significantly inhibited tumor growth and improved survival than either drug alone [102]. The epigenetic modifier EZH2 has also been shown to suppress IFN-y induced PDL-1 expression in HCC cell lines, thus representing a possible therapeutic target for inhibition and subsequent combination with anti-PD-1/PDL-1 therapy [103]. Reduction of EZH2 expression in Tregs has also translated into improved efficacy of anti-CTLA-4 therapy [104].

Several clinical studies of checkpoint inhibitors combined with epigenetic modifiers, including IDO inhibitors which have also demonstrated similarly favorable immunomodulatory effects pre-clinically [105], are currently underway in GI cancers (Table 4). A recently reported pilot study of ‘priming’ with either 5-AZA or romidepsin (HDACi) or the combination of both, followed by administration of pembrolizumab to patients with MSS mCRC established that the combination of these agents with checkpoint blockade was safe and tolerable [106]. It will be of interest to see the corresponding translational results from the sequential biopsies which were taken pre- and post- prime as to whether the TME was indeed favorably sensitized for subsequent checkpoint inhibitor therapy, consistent with the pre-clinical data.

### Combination checkpoint blockade and Wnt signaling modulators

Abberations in the Wnt/β-catenin signaling pathway occur frequently in cancer and while the constituents of the pathway are known, the mechanisms underpinning how the proteins interact are yet to be fully elucidated [107]. The role of Wnt signaling in development of colorectal cancer, most commonly via mutations of the APC gene, is particularly well-described however increasing evidence suggests that Wnt signaling also plays a key role in the pathogenesis of other GI cancers including PDAC, HCC, CCA and OG cancers [107–110], proposing a potential target for therapy.

Preliminary clinical studies of Wnt signaling modulators as monotherapies including Porcupine inhibitors, Frizzled receptor targeting agents, a Wnt5a-mimetic and agents targeting components further downstream in the pathway, have so far demonstrated promising safety profiles in patients with GI cancers where side effects including bone toxicity appear to be manageable [111–116], however signals of single agent efficacy have yet to be confirmed. In parallel, mounting evidence indicates a critical role for Wnt/β-catenin signaling in immunomodulation of the TME at multiple steps of the Cancer Immunity Cycle [117]. and thus the possibility of combining Wnt modulators with immune checkpoint inhibitors has emerged as an attractive therapeutic strategy (Fig. 2).

Firstly, activated Wnt/β-catenin signaling appears to accentuate tumor immune exclusion by suppressing DC recruitment into the TME via down-regulation of the chemokine CCL4 as well as impaired priming of effector T cells in melanoma mouse models, which were subsequently resistant to immune checkpoint blockade [118, 119]. Successively, activated Wnt/β-catenin signaling has also been shown to drive development of a non-T cell inflamed TME in other tumor types including GI cancers, most notably, esophageal, HCC and CRC [120, 121]. By inactivating Wnt/β-catenin signaling, presentation of cancer associated antigens and T cell priming appears to be restored [122]. In addition to impairing the first 3 steps of the Cancer Immunity Cycle, aberrant Wnt/β-catenin signaling also seems to deter T cells from entering the tumor and preferentially favors influx and survival of inhibitory Tregs while inactivating or stimulating apoptosis of effector T cells in the immune milieu [123, 124]. Metastatic lung and breast tumors also seem to be able to evade immune detection by secreting the Wnt inhibitor DKK1 in an autocrine manner [125] and elevated levels of DKK1 appear to be associated with worse prognosis in OG cancer [126]. DKK1 has since been shown to increase tumor growth and support an immunosuppressive environment by signaling to MDSCs, and treatment with a DKK1 neutralizing antibody, DKN, 01, was able to mitigate tumor growth by reduced levels of MDSCs and increased entry of effector T cells into the TME [127, 128]. These results suggest that DKN-01 may favorably reprogram the immune milieu for collaboration with checkpoint inhibitors.

In the clinic, the combination of DKN-01 and pembrolizumab has been shown to be safe and tolerable in patients with advanced MSS OG cancer with a hint of potential efficacy as a PR was observed in 1 patient and 5 patients had SD [129]. Interestingly, these 6 patients also had a reduction of MDSC levels in peripheral blood on treatment compared to baseline, consistent with pre-clinical observations [129]. Another clinical study of DKN-01 and atezolizumab will similarly assess the safety and efficacy of combination Wnt inhibition and checkpoint blockade and studies of combined Porcupine inhibitors and ICIs are also currently recruiting (Table 4).

### Combination checkpoint blockade and stromal targeting agents

The tumor stroma is comprised of dense connective tissue including CAFs, stromal cells, osteoblasts, chondrocytes and ECM and it is a critical component of the TME [130]. Activated desmoplastic stroma is particularly unique to PDAC and is a huge barrier to effective delivery of anti-cancer therapy including immunotherapy [36, 130]. Thus, potential combination strategies involving stromal targeting agents and checkpoint inhibitors have recently garnered increased attention in this disease.

High levels of focal adhesion kinases (FAK) in PDAC appear to correlate with low levels of CTLs and a generally immunosuppressive TME and in mouse models FAK inhibition (FAKi) appeared to reduce tumor fibrosis and halt PDAC progression as well as decrease populations of immunosuppressive cells such as TAMs and Tregs [131]. FAKi also demonstrated increased T cell infiltration and promoted tumor shrinkage with addition of ICI suggesting a potentially efficacious synergy [131]. A clinical study of defactinib (FAKi) and pembrolizumab is currently underway in patients with solid tumors including PDAC (Table 4).

Agents targeting chemokine proteins and chemokine receptors which are involved in cell migration have also demonstrated potential as valuable ICI partners in PDAC. For example, the chemokine CXCL12 secreted by CAFs and the chemokine receptor CXCR4 found on T cells appear to drive immunosuppression in the TME by increasing populations of MDSCs [132, 133]. CXC12 inhibition combined with checkpoint blockade showed improved T cell infiltration and halted tumor growth in mouse models [133] and the phase II COMBAT trial of combination CXCR4 blockade with BL-8040 plus pembrolizumab recently reported a DCR of 34.5% in an ITT of 29 patients and mOS of 7.5 months as a second-line therapy in patients with advanced PDAC [134]. Supportively, the corresponding translational analyses indicated that BL-8040 reduced MDSC and Treg populations and increased tumor infiltration with CTLs [134]. These exciting results are likely to lead onto confirmatory phase III studies. Preliminary results from the safety run of mogamulizumab, a CCR4 antibody, in combination with nivolumab revealed confirmed tumor responses in patients with HCC and PDAC and similar immunomodulatory effects on the TME which also holds promise [135]. Additionally, CXCR2 and CCR2 or CSF1R inhibition have been shown to improve T cell infiltration, reprogram TAMs, upregulate PD-L1 and CTLA-4 checkpoints and increase sensitivity to ICIs in PDAC mouse models [136–138] which has led onto further assessment of these combinations in clinical studies (Table 4).

Alternative combination approaches include use of Bruton’s tyrosine kinase (BTK) inhibitors, which have been shown to deplete MDSCs in the TME, improve CTL activity and ameliorate the fibrous stroma of PDAC pre-clinically [139–141] with ICIs. However while the phase II study of the BTK inhibitor, alacabrutinib, combined with pembrolizumab showed reduction of MDSCs in peripheral blood from patients on study and the combination was tolerable, the response rates were limited [142]. Thus highlighting the complexity of the TME in PDAC. More promising strategies in the pipeline may include partnering CD40 agonists which favorably alter the immune component of the PDAC stroma [143]. with chemotherapy and PD-1 blockade (Table 4) and use of the bispecific antibody, M7824, which blocks both immune-inhibitory TGFβ and PD-L1 pathways and has demonstrated some efficacy signals in patients with PDAC and BTC [144, 145].

## Conclusion

Over the past few years, data supporting clinical benefit for immune checkpoint inhibitors in the treatment of gastrointestinal cancers has gradually mounted and approvals for specific indications have come through. However, the challenge of expanding the benefit of immunotherapy to the majority of the population with non-T cell inflamed, ‘cold’ gastrointestinal tumors with intrinsic resistance to these therapies is still very much an unmet need. Research efforts into effective predictive biomarker identification, fraught with hurdles such as intratumoral heterogeneity, is still being explored and refined however much headway has already been made. Furthermore, rational combinatorial strategies of immune checkpoint inhibitors with agents that produce beneficial immunomodulatory effects and reprogram the TME to overcome intrinsic resistance to effective anti-cancer immunity, hold significant promise. The corresponding translational components of the clinical studies discussed herein may be the key to gaining a deeper understanding of underlying mechanisms of response and resistance to these strategies and slowly unlock more about the intricacies of the immune milieu in these malignancies with the hope of discovering more efficacious treatments for our patients.
